# QIP Improving Trainee Confidence in Male Sexual Dysfunction History-Taking in an Acute Inpatient Unit

**DOI:** 10.1192/bjo.2024.383

**Published:** 2024-08-01

**Authors:** Alice Humphrys, Rahul Mehta

**Affiliations:** West London NHS Trust, London, United Kingdom

## Abstract

**Aims:**

Trainees on the psychiatry on-call rota at a London acute inpatient unit reported a lack of confidence in asking male patients about sexual dysfunction during clerking. Research shows that history-taking barriers include embarrassment, time shortage and task prioritisation. Sexual dysfunction is prevalent amongst the general population, markedly so amongst people with mental health diagnoses.

In response, we designed a quality improvement project (QIP) to improve confidence by addressing the need for good history-taking and the technique for doing so.

**Methods:**

To gauge trainee confidence, we produced and disseminated an online questionnaire with a mixture of qualitative and quantitative questions.

Based on the data collected, we contacted a local sexual health consultant and requested a teaching session on the importance of sexual history-taking, the impact of not doing so, barriers to history-taking and how to ask about sexual dysfunction.

A follow-up questionnaire was produced and disseminated.

**Results:**

The results of the first questionnaire showed that 100% of respondents (n = 10) did not ask male patients questions about their sexual function, on admission. The main reasons for this were embarrassment for themselves (25%) and the patient (66.7%), lack of confidence on how to word these questions (50%), lack of time (58.3%) and feeling that these questions are not relevant (33.3%).

Following the teaching session, 71.4% of respondents said that they would ask male patients questions about symptoms of sexual dysfunction on admission. The majority of responses quoted that the teaching had increased their confidence, decreased their embarrassment in asking these questions, and helped them to understand the relevance of asking these questions. Two respondents queried the appropriateness of asking acutely unwell patients these questions on admission and if these questions could be asked during a patient's admission instead.

*Limitations*: Small sample size of results; slight drop in responses from first questionnaire to second questionnaire; questionnaire only asking questions about male patients, not female patients.

**Conclusion:**

This QIP shows that a single, simple intervention can improve trainee confidence in the short term. This intervention can be applied across the UK. Online teaching can improve access to the expertise of local sexual health consultants. This QIP also provides a basis for further analysis: whether single interventions can improve trainee confidence in the long term, when is the best time to ask questions about sexual function and applying this intervention to female sexual function history-taking.

